# Characterization of different adipose depots in fattened buffalo: histological features and expression profiling of adipocyte markers

**DOI:** 10.5194/aab-63-61-2020

**Published:** 2020-02-21

**Authors:** Jieping Huang, Xiaoyan Liu, Xue Feng, Mingming Zhang, Kaixing Qu, Jianyong Liu, Xuefeng Wei, Bizhi Huang, Yun Ma

**Affiliations:** 1 College of Life Sciences, Xinyang Normal University, Xinyang, Henan, 464000, China; 2 Academy of Grassland and Animal Science, Kunming, Yunnan, 650212, China; 3 School of Agriculture, Ningxia University, Yinchuan, Ningxia, 750021, China

## Abstract

Adipose tissue (AT) is a multi-depot organ in mammals. AT from various depots differs in composition and function. Revealing the composition feature of AT depots will provide valuable information for further research on the development and fat deposition patterns in buffalo. This study explored the cellular morphology and gene expression profiles of brown and beige markers in seven AT depots of fattened buffalo: three subcutaneous depots (back, sternum, and inguinal) and four visceral depots (perirenal, mesenteric, pericardial, and omental). Histological results showed unilocular adipocytes in all seven AT depots. Uncoupling protein 1 (*UCP1*) mRNA, a brown and beige adipocyte gene, was detected in all depots with the highest level in VAT depots, and a limited number of UCP1-positive unilocular adipocytes were observed in the three VAT depots. The mRNAs of PPARG coactivator 1 alpha (*PGC1* α) and transmembrane protein 26 (*TMEM26*), brown or beige adipocyte markers, were identified in all seven depots and were mainly expressed in VAT depots. However, the mRNA of zinc finger protein of the cerebellum 1 (*ZIC1*), a brown adipocyte-specific marker, was almost undetectable. Our results demonstrated that all seven AT depots are white adipose tissue (WAT), with potential function of non-shivering thermogenesis in fattened buffalo. Beige adipocytes are more active in VAT depots than in WAT depots. These results improve our knowledge on the feature of different adipose tissue depots in buffalo, which will be useful for the research of fat deposition.

## 1 Introduction

Adipose tissue (AT) in mammals is a multi-depot endocrine organ that secretes numerous humoral factors to regulate multiple biological processes, including energy metabolism. AT is formed in specific locations at specific times to meet the requirements of organism development. AT depots from various locations differ in morphology, composition, and function, and it has been suggested that each AT depot should be considered a different tissue (Kruglikov and Scherer, 2016). There are two main regional AT depots in mammals: subcutaneous (SAT) and visceral (VAT). The SAT and VAT depots have different gene expression profiles, indicating that they are biologically distinct (Atzmon et al., 2002; Vohl et al., 2012). In humans, SAT is associated with insulin sensitivity, type II diabetes, and other metabolic disorders (Misra et al., 1997; Snijder et al., 2004), and VAT is associated with insulin resistance and dyslipidemias (Wajchenberg et al., 2002), type II diabetes (Boyko et al., 2000), hypertension (Hayashi et al., 2003), and all-cause mortality (Kuk et al., 2006). AT depots can be classified as white AT (WAT) and brown AT (BAT), and these tissues differ in both morphology and function. Morphologically, WAT is characterized by containing unilocular lipid droplet, while BAT is rich in small lipid droplets (Cinti, 2005). Functionally, WAT is considered to be a storage site of excess energy, whereas BAT is primarily responsible for non-shivering thermogenesis. In recent studies, a new type of adipocyte has been described: beige adipocytes, the morphology and function of which are in between those of white and brown adipocytes.

AT has a significant role in maintaining organism health. It has been the focus of many animal studies, and as a result there is a relatively clear understanding of the morphology and composition of different AT depots in humans and rodents (Wu et al., 2012). SAT in the cervical, supraclavicular, and subscapular regions are considered to be BAT in humans (Cypess et al., 2013; Lidell et al., 2013); in rodents five depots have been identified as BAT: interscapular, cervical, axillary, mediastinal, and perirenal (Waldén et al., 2011). In bovines, BAT has been found in fetal cattle (Taga et al., 2012), and an abundance of BAT markers have been detected in multiple WAT depots of fattened cattle (Asano et al., 2013; Komolka et al., 2017). To date, there have been no studies on the brown and beige adipocytes found in *Bubalus bubalis* (common buffalo), a species of bovine that plays a vital role in beef production in several southeastern and Middle Eastern Asian countries and Africa (Naveena and Kiran, 2014).

By burning chemical energy to produce heat, brown and beige adipocytes counteract obesity and metabolic disease in humans or rodents. In bovines however, brown and beige adipocytes either act as a heat producer to improve the capacity of cold resistance (Trayhurn, 2009) or they may have a negative effect on feeding efficiency (Asano et al., 2013). As BAT markers have been identified in the WAT depots in fattened cattle (Asano et al., 2013; Komolka et al., 2017), we hypothesized that similar markers exist in buffalo too. To reveal the composition and potential function of different AT depots in fattened buffalo, seven AT depots were resected for morphological analysis by hematoxylin and eosin staining (H&E), as well as RT-qPCR analysis of brown and beige adipocyte gene expression and immunohistochemistry. This study will make an important contribution to our current understanding of the composition and differences of AT depots in fattened buffalo.

## 2 Materials and methods

### 2.1 Animal ethics

Buffalo were bred for commercial use instead of experimental reasons and were slaughtered by a Muslim cleric according to Islamic law. Thus, no ethics approval was required by a specific committee.

### 2.2 Animals and sampling

**Figure 1:**
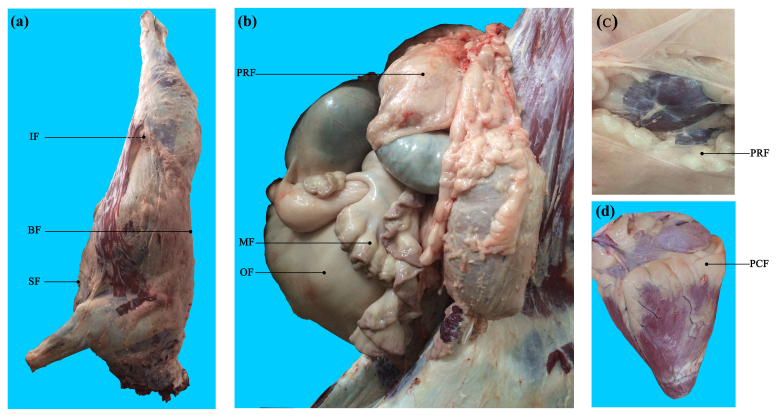
Visualization of adipose tissue depots sampled in buffalo. **(a)** Carcass of a Binlangjiang buffalo. **(b)** The abdomen was opened up to expose the internal organs. **(c)** The perirenal fat was peeled off to expose the kidney. **(d)** The heart. A total of seven depots were sampled. BF, back fat; SF, sternum fat; IF, inguinal fat; PRF, perirenal fat; MF, mesenteric fat; PCF, pericardial fat; OF, omental fat. The same abbreviations are used in Figs. 2 and 3.

Binlangjiang buffalo (n=6) were raised according to the standard conditions of the Tengchong Buffalo Farm (Tengchong, Yunnan, China). Buffalo were fattened at 12 months old and were slaughtered at 24 months old in the slaughterhouse of Yunnan Academy of Grassland and Animal Science (Kunming, Yunnan, China). The temperature of the slaughter house was approximately 23 ${}^{\circ }$C. For each buffalo, seven AT depots, including three subcutaneous AT depots (back, sternum, and inguinal) and four visceral AT depots (perirenal, mesenteric, pericardial, and omental) (Fig. 1), were sampled after slaughter and immediately frozen in liquid nitrogen. Obvious fascia and vessels in AT were removed before sampling.

### 2.3 Adipose tissue histology

The AT samples were fixed in 4 % formaldehyde overnight at 4 ${}^{\circ }$C. Then, the tissue was dehydrated in ethanol, cleared in xylene, and embedded in paraffin. The sample was sectioned with a 4 µm thickness. The sections were then mounted on clean slides, dewaxed with xylene, and washed with an ethanol gradient to water. For light microscopy, sections were stained with H&E. The section was fixed with hematoxylin staining solution for 10 min, washed with water, treated with alcohol hydrochloride differentiation solution for 3 s, washed with water, and treated with Scott solution for 3 min. The section was then counterstained with eosin for 10 min, washed with ethanol, treated with xylene, and sealed with neutral resin. A Nikon Eclipse Ci series microscope equipped with a digital camera system was used for imaging.

A rabbit polyclonal antibody against human uncoupling protein 1 (UCP1) (ab10983, Abcam, Cambridge) was used for immunohistochemistry (Asano et al., 2013). After dewaxing and washing with water, the section (4 µm thickness) was fixed with sodium citrate buffer at 100 ${}^{\circ }$C for 20 min. To block the endogenous peroxidase, the section was then treated with 0.3 % $H_{2}O_{2}$ in methanol for 20 min at room temperature and washed with phosphate buffer saline (PBS) three times. Goat serum (2 %) was used as a blocking solution to fix the section for 30 min, which was then washed with PBS three times. Then, the section was incubated with the anti-UCP1 antibody (diluted 1:200) overnight at 4 ${}^{\circ }$C and washed with PBS three times. The section was then incubated with polymer adjuvant at 37 ${}^{\circ }$C for 20 min. The secondary antibody was added and incubated for 2 h at room temperature. Then, the sections were washed with PBS, colored with a DAB substrate kit, and counterstained with hematoxylin. Finally, the section was dehydrated and mounted.

### 2.4 RNA isolation and RT-qPCR

Total RNA was extracted using TRIzol (Invitrogen, USA) according to the manufacturer's instructions. Total RNA was reverse-transcribed into cDNA using a PrimeScriptsRT reagent kit with a gDNA Eraser (TaKaRa, Japan). qRT-PCR was performed in triplicate using SYBR Green I (TaKaRa) with two-step reactions. The values were normalized to the expression of *IQGAP1* and *UXT* for each sample, and the fold change was determined by $2^{-\Delta \Delta Ct}$. Details of the primers are presented in Table 1. The PCR products were detected by agarose gel electrophoresis to document the quality of qRT-PCR (Fig. S1 in the Supplement).

**Table 1:** Details of the primers used for the RT-qPCR assay.

Gene	Forward primer (5${}^{\prime }$–3${}^{\prime }$)	Reverse primer (5${}^{\prime }$–3${}^{\prime }$)	Product	Tm
			(bp)	(${}^{\circ }$C)
*UCP1*	AAACAGAAGGGCCAGTGAAA	TGCAGTCTGACCTTGACCAC	220	60
*ZIC1*	AAGGTCTTCGCCCGTTCT	TAGGGCTTGTCGCTTGTATG	155	59
*PGC1*α	ACCTCCATTTTTGAGCATCAG	ACGCGCCAAACTTTACTGAC	74	63
*TMEM26*	GCGCTGCTTAATCTCTTGCT	TGCAATACTGGGTTTCATGGT	160	61
*IQGAP1*	AAGAAGGCGTACCAAGACCG	GTGCATCCTTGCCAGAGACT	84	60
*UXT*	GTTGACACAGTGGTCCCAGA	ATGTCAGGGGAGGTAGGAAGAA	260	60

### 2.5 Statistical analyses

Data were analyzed by SPSS 19 software. For significant difference analysis, a one-way ANOVA followed by Tukey's test was performed. A value of p<0.05 was considered statistically significant. The results were presented as mean ± SEM by Origin™ program.

## 3 Results and discussion

### 3.1 Morphology analysis of AT depots

**Figure 2:**
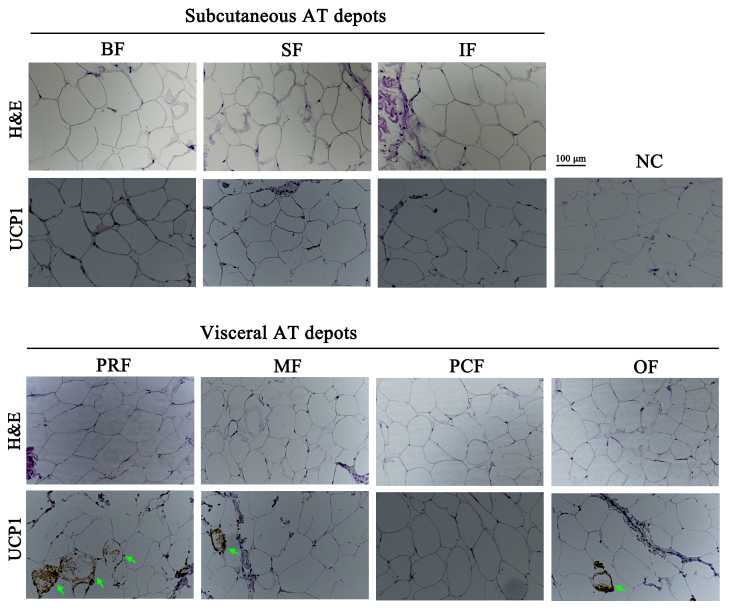
H&E staining and immunolocalization of *UCP1* in adipose tissue depots of fattened buffalo. Cellular morphology of the seven adipose tissue depots examined by H&E staining. An immunohistochemistry assay identified UCP1-positive cells (green arrow). NC, negative control. Scale bar, 100 µm.

Adipocyte morphology can be used to characterize AT type. The morphological difference between white and brown adipocytes can be identified with H&E staining (Lidell et al., 2013; Cypess et al., 2013; Matthew et al., 2016). In this study, unilocular adipocytes, the classical morphological indicator of white adipocytes, were seen after H&E staining in all seven AT depots (Fig. 2), which suggests that they are WAT depots.

In humans and rodents, most AT depots are WAT, with BAT limited to specific depots in babies and adults (Chusyd et al., 2016). In bovines, adipocytes containing multilocular lipid droplets are enriched in animals at 110 d post-conception, but levels significantly decrease as they age (Taga et al., 2012) and can completely disappear after 30 d post-birth (Alexander et al., 1975). To the best of our current knowledge, no classical brown adipocytes have been identified in adult bovines. The morphological results presented here suggest that the back, sternum, inguinal, perirenal, pericardial, omental, and mesenteric AT depots in fattened buffalo are all WAT.

### 3.2 Gene expression of brown and beige adipocyte markers

In WAT, white adipocytes can be converted into beige adipocytes by environmental or intrinsic factors (Wu et al., 2012). Although all seven AT depots examined here showed white adipocyte morphology, other adipocyte types may also be present. According to studies in rodents and humans, a specific collection of genes can be used as brown and beige adipocyte markers (Waldén et al., 2011; Wu et al., 2012; Cypess et al., 2013). In our study, three brown adipocyte markers – *UCP1*, zinc finger protein of the cerebellum 1 (*ZIC1*), and PPARG coactivator 1 alpha (*PGC1* α) – and a beige adipocyte marker – transmembrane protein 26 (*TMEM26*) – were used to reveal any brown and beige adipocyte populations in the seven AT depots.

#### 3.2.1 *UCP1*

**Figure 3:**
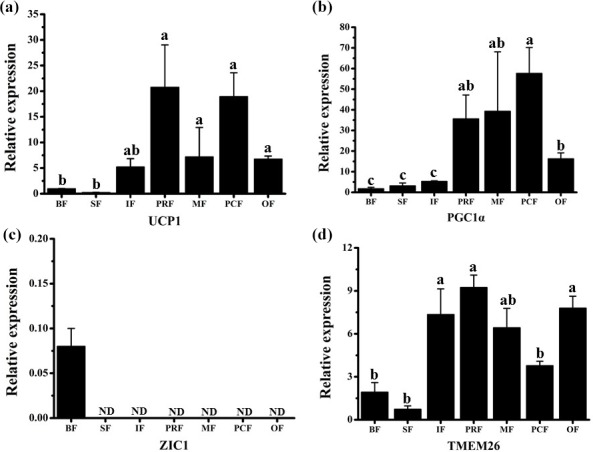
Expression profile of adipocyte markers in adipose tissue depots of fattened buffalo. The relative mRNA expression levels of four adipocyte markers, *UCP1* **(a)**, *PGC1* α **(b)**, *ZIC1* **(c)**, and *TMEM26* **(d)**, analyzed by RT-qPCR. Expression levels were normalized to β-actin. Data are shown as the mean ± SEM (n=6). Different lowercase letters (a–c) indicate significant difference (p<0.05) between depots. ND, not detectable.

UCP1 was previously believed to be a unique indicator for brown adipocytes. However, in recent studies, a new type of adipocyte has been described: beige adipocytes, which express *UCP1* but have a gene expression pattern distinct from brown adipocytes (Wu et al., 2012). The presence of *UCP1* in WAT is one of the first indicators of beige adipocyte existence (Rossmeisl et al., 2002). Therefore, the *UCP1* detected here in the WAT of fattened buffalo suggests that AT is made up of a heterogeneous population of adipocyte types (Fig. 3a). We found that *UCP1* mRNA was detected in all depots by RT-qPCR and was mainly expressed in the VAT depots (Fig. 3a, p<0.05). There was considerable expression in the inguinal depot, a deeper SAT depot. In addition, we also detected UCP1 using immunohistochemistry. However, only few UCP1 unilocular adipocytes had a scattered distribution in three of the four VAT depots, including perirenal, mesenteric, and omental (Fig. 2). The UCP1-positive adipocytes were richer in the perirenal depot than in other depots (Fig. 2). No UCP1-positive adipocyte was found in other four depots; this might be caused by the fact that beige adipocytes were not evenly distributed in the depots as mentioned in a previous study (Komolka et al., 2017). In others, the UCP1-positive adipocytes might be too limited to be detected in other four depots.

In mice, the perirenal AT is considered to be BAT (Waldén et al., 2011). In bovines, *UCP1* is detectable in the perirenal depot at 180 d post-conception (Taga et al., 2012), and its levels increase until birth (Casteilla et al., 1987, 1989; Smith et al., 2004). It then disappears quickly and cannot be detected later than two days after birth (Casteilla et al., 1989). Similar variations have been observed in lambs (Casteilla et al., 1989). In adult bovines, *UCP1* has been widely detected in SAT and VAT depots (Asano et al., 2013; Komolka et al., 2017). A study on fattened cattle observed a relatively high expression level of *UCP1* mRNA in perirenal depots and UCP1-positive adipocytes in WAT (Komolka et al., 2017). In this study, the average of *UCP1* mRNA expression is highest and UCP1-positive adipocytes is richest in perirenal depots. These lines suggest that there are different adipocyte types in the WAT of fattened buffalo. UCP1 diverts energy from ATP synthesis to thermogenesis in the mitochondria of brown adipocyte. Thus, WAT, especially the perirenal fat, is for more than the storage of excess energy in fattened buffalo.

#### 3.2.2 *PGC1* α and *ZIC1*

As *UCP1* was detected in all seven AT depots, we wanted to see if other BAT markers were expressed as well. The mRNAs of both *PGC1* α and *ZIC1* were detected to confirm the presence of BAT. We found that *PGC1* α mRNA was expressed in all depots. The highest level was detected in three VAT depots (perirenal, mesenteric, and pericardial), followed by other VAT depot (omental) (Fig. 3b). *ZIC1* mRNA however, was detected in only back depot (Fig. 3c). These results were consistent with those in a previous study (Komolka et al., 2017). In brown adipocytes, PGC1α controls thermogenic gene activation (Lin et al., 2004; Uldry et al., 2006), whereas in white adipocytes the expression of PGC1α turns on the characteristics of brown adipocytes, up-regulating the expression of UCP1 in mitochondria (Puigserver et al., 1998; Tiraby et at. 2003). By contrast, *ZIC1* expression has been shown to be specific for BAT depots (Waldén et al., 2011). These results further suggest that there is no brown adipocyte in the seven AT depots. Beige adipocytes may exist, especially in the VAT depots.

#### 3.2.3 *TMEM26*

*TMEM26* has been identified in several studies as a beige adipocyte marker (Cypess et al., 2013; Lidell et al., 2013; Xue et al., 2014). In a previous study, *TMEM26* expression was widely detected in multiple depots in cattle (Komolka et al., 2017). We found that *TMEM26* was detectable in all seven depots and was mainly expressed in the VAT and the inguinal depots (Fig. 3d), which was similar to the expression pattern of *UCP1*. In fact, the inguinal depot is positioned deeper than back and sternum depots. In humans, beige adipocytes are richer in the deep neck fat than the subcutaneous neck depots in adults (Cypess et al. 2013). To sum up, *ZIC1* mRNA was detected in only back depot with a very low expression level. *UCP1*, *PGC1* α, and *TMEM26* mRNAs were detectable in all seven depots. Importantly, the expression levels of *UCP1*, *PGC1* α, and *TMEM26* in VAT depots were almost higher than SAT depots, especially than the back and sternum depots. These results indicate that beige adipocytes, rather than brown adipocytes, exist in the WAT depots, especially in VAT or deep depots of fattened buffalo. Beige adipocytes can be induced by body mass index (Timmons and Pedersen, 2009), insulin sensitivity (Timmons and Pedersen, 2009), cold acclimation (Barbatelli et al., 2010), and a high-energy diet (Asano et al., 2013). In this study, buffalo were fattened on a high-energy diet for 1 year and not exposed to the cold before slaughter. Therefore, beige adipocytes in WAT depots should be induced by a high-energy diet in our fattened buffalo. Thus, this negative effect on feeding efficiency should be considered in fattened buffalo. It should be noted that our results suggest that beige adipocytes are more active in VAT or deep depot than those in SAT depots, which is different from those results in cattle (Asano et al., 2013; Komolka et al., 2017). This could be a real difference between buffalo and cattle or the difference in sampling. Bovine is a large livestock animal and has a long breeding cycle, which make the feeding and management conditions in various studies difficult to unify. In addition, AT can be widely distributed throughout the body, resulting in sample difference. Thus, studies with more strict management conditions and a higher number of samples are needed to confirm the difference between buffalo and cattle.

## 4 Conclusions

To our knowledge, this is the first study on the composition of different AT depots in fattened buffalo. We have shown that (1) all seven AT depots examined (back, sternum, inguinal, perirenal, mesenteric, pericardial, and omental) are WAT; (2) beige adipocytes markers are widely expressed in WAT; and (3) beige adipocytes are more active in VAT depots. These results suggest that a negative effect on feeding efficiency by beige adipocytes should be considered in fattened buffalo.

## Supplement

10.5194/aab-63-61-2020-supplement The supplement related to this article is available online at: https://doi.org/10.5194/aab-63-61-2020-supplement.

## Data Availability

The original data are available upon request from the corresponding author.
