# Supplementary material for: Characterization of different adipose depots in fattened buffalo: histological features and expression profiling of adipocyte markers
Source: Arch Anim Breed. 2020 Feb 21;63(1):61–7. doi: 10.5194/aab-63-61-2020 (PMC7059603; doi:10.5194/aab-63-61-2020)
Supplement: The supplement related to this article is available online at: https://doi.org/10.5194/aab-63-61-2020-supplement. [file aab-63-61-supplement.pdf]

Supplement of Arch. Anim. Breed., 63, 61–67, 2020
https://doi.org/10.5194/aab-63-61-2020-supplement
© Author(s) 2020. This work is distributed under
the Creative Commons Attribution 4.0 License.

Open Access
Archives Animal Breeding

Supplement of

Characterization of different adipose depots in fattened buffalo: histological features and expression profiling of adipocyte markers

Jieping Huang et al.

Correspondence to: Yun Ma (mayun_666@126.com) and Bizhi Huang (hbz@ynbp.cn)

The copyright of individual parts of the supplement might differ from the CC BY 4.0 License.

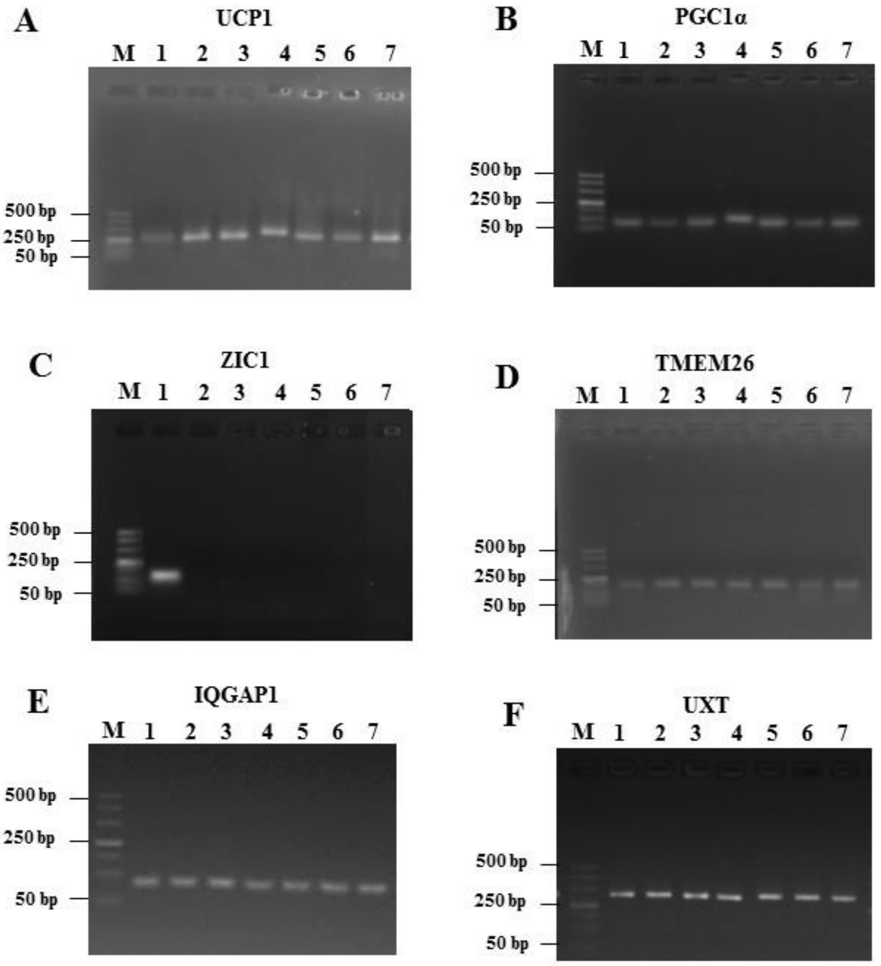

Figure S1 Detection of qRT-PCR products by agarose gel electrophoresis. M, Marker 500. 1-7, back, sternum, inguinal, perirenal, mesenteric, pericardial, and omental fats, respectively.
